# First molecular detection of porcine rotavirus B (RVB) in Poland – case study and genome analysis

**DOI:** 10.1186/s40813-025-00472-3

**Published:** 2025-11-13

**Authors:** Piotr Cybulski, Ines Spiekermeier, Weronika Rybkowska, Jędrzej Rynkowski, Tomasz Stadejek

**Affiliations:** 1 Goodvalley Agro S.A, Dworcowa 25, Przechlewo, 77-320 Poland; 2 SAN Group Biotech Germany GmbH, Mühlenstrasse 13, 49685 Höltinghausen, Germany; 3 Department of Pathology and Veterinary Diagnostics, Institute of Veterinary Medicine, Warsaw University of Life Sciences−SGGW, Nowoursynowska 159C, Warsaw, 02-776 Poland

**Keywords:** Pigs, Porcine rotavirus b, Piglet diarrhoea, Phylogenetic analysis

## Abstract

**Background:**

In view of its massive financial impact related to increased preweaning mortality and poor growth performance, neonatal diarrhoea in piglets is widely regarded as one of the most common issues in modern swine production. Currently, the diarrhoea of undetermined aetiology arises as a complex diagnostic problem in several pig-rearing countries; therefore, the role of agents with not completely established clinical importance, such as non-group A rotaviruses (RVs), should be taken into consideration in a differential diagnosis procedure. The aim of this study was to report the detection of RVB acting as a potential causative agent of porcine diarrhoea.

**Results:**

The investigation was conducted in a Polish commercial, high-performing farm with 3,300 sows weaning piglets in weekly batches. For the scope of this research, five 3-day-old dead diarrhoeic piglets were collected. All the animals originated from different litters displaying clinical signs regarded as typical of rotaviral enteritis. Prior to the NGS investigation, all the piglets sampled in our current investigation were tested negative for diarrhoea-associated viruses (RVA, RVC, TGEV, PEDV) using commercially available PCR setups. The NGS data for the faecal sample resulted in 510,843 reads after adapter trimming. 981 reads were assigned at species level to RVB (strain GCZ04) during read classification. The genotype constellation of the strain was found to be typical for swine viruses and the nucleotide identities of the segments of the strain were within the cut off values established for different genotypes. The remaining viral reads were annotated to porcine kobuvirus (PKV) (23 reads). Furthermore, the analysis revealed presence of the following taxa: Qubevirus faecium (334 reads), and Gaprivervirus (1 read).

**Conclusions:**

Our study reports the first molecular detection of porcine RVB in faecal samples collected from pigs reared on a commercial swine farm in Poland. Since the material was obtained from clinically affected animals and RVB was proven dominant among viral reads obtained during the NGS investigation, the virus may be considered as a potential causative agent of diarrhoeal disease in suckling piglets. Moreover, our research provides the tenth porcine RVB genome in Europe. The identities and phylogenetic clustering of different segment sequences to those from North America, Asia or Europe may suggest a complex evolutionary history of the Polish strain.

## Background

In view of its massive financial impact related to increased preweaning mortality and poor growth performance, neonatal diarrhoea in piglets is widely regarded as one of the most common issues in modern swine production. Besides the level of passive immunity provided to neonates and influence of broadly understood environmental conditions, the incidence of the disease can be very much affected by a set of numerous contagious factors which have been identified over the years [1]. Historically, different pathotypes of *Escherichia coli*, *Clostridium perfringens*, *Clostridioides difficile*, rotavirus (RV) type A and C, porcine epidemic diarrhoea virus (PEDV) and *Cystoisospora suis* were regarded as the most important agents causing the disease. Currently, neonatal diarrhoea of undetermined aetiology arises as a complex diagnostic problem in several pig-rearing countries; therefore, the role of agents with not completely established clinical importance, such as non-group A rotaviruses, should be taken into consideration in a differential diagnosis procedure.

Since their first description in humans in the early 1970s [2], RVs have been recovered from faecal samples in a remarkable variety of hosts and remain a significant cause of severe diarrhoeal illness in humans and animals [3, 4]. The infection spreads *via* faecal-oral exposure and poses a considerable threat to public health. Affecting young children and infants in both developing and developed countries, RVs are estimated to be responsible for approximately 108,000 deaths occuring annually in children under five years of age [5]. From the veterinary perspective, RVs cause considerable financial losses in modern animal farming, emerging as a factor of diarrhoea in suckling piglets [1], calves [6], foals [7], and lambs [8].

RVs belong to the genus *Rotavirus* within the family *Sedoreoviridae* and retain biological features typical of all the family members. RVs are characterised by cytoplasmic replication, mutation of individual genes (random genetic drift), as well as genetic reassortment occuring within each serogroup or genus (antigenic shift). Morphologically, the mature RV particle resembles a wheel and is non-enveloped, with 11 double-stranded RNA (dsRNA) genome segments (assigned 1–11), each coding for at least one viral protein: six structural (VP1-VP4, VP6, VP7) and five non-structural ones (NSP1-NSP5), including NSP4, described as the very first viral secretory enterotoxin [9]. The virion is complex and triple-layered. Consisting of three proteins (VP1-VP3), the core is covered by the intermediate layer composed of VP6 subunits. The outer capsid forming a nearly regular spherical icosahedron comprises of VP7. Multiple VP4 dimers spiking from the particle surface span the outer protein shell and the VP6 layer. Since the latter protein is antigenically conserved, its sequence and antigenic differences serve as a diagnostic target allowing subdivision of RVs into 11 distinct species (RVA-RVD, RVF-RVL) [10–12]. Within the species, RVs can be further classified into serotypes based on VP4 and VP7 epitopes [13].

The pathogenesis of RV diarrhoea is similar in virtually all species studied to date and generally clinical manifestation related to the digestive track remains the most prominent [14]. After the ingestion, the virions pass to the small intestine, infect and replicate in enterocytes, mainly in the jejunum and ileum, generating cell exfoliation and villi atrophy [13]. Consequently, poorly absorbed solutes in the intestinal lumen cause osmotic diarrhoea. In addition to nutrient and electrolyte malabsorption promoted by the above-described alterations, the mechanisms of secretory rotaviral diarrhoea have also been elucidated, pointing to the biological activity of NSP4, recognised as a viroporin increasing levels of intracellular Ca^2+^ [9].

The RV infections were described in nearly all species of domestic animals [13]. While the majority of RVs seem to be host-specific, several cases of interspecies transmission have been reported [15]. To date, four serotypes of RVs have been detected in swine (RVA, RVB, RVC, RVH); however, only three of them, i.e. RVA, RVB, and RVC have been validly proven to cause a diarrhoeal disease in swine [1]. Since its first isolation in 1975 [16], RVA has been considered the most prevalent and pathogenic member of the genus *Rotavirus* in pigs worldwide [3]. Porcine RVC was originally described in a 27-day old diarrhoeic pig reared in the USA [17]. Similarly to RVA, the virus has been identified worldwide as a cause of diarrhoea in young pigs, either as a monoinfection or with coinfecting RVs [18–21]. In the USA, Canada and Mexico 51.1% (3833/7508) of intestinal samples collected from pigs were found RVC-positive [22]. Subsequent to its first identification in the 1980s [23], the knowledge regarding porcine RVB remains fairly limited. Unlike RVA, pathogenesis of RVB-associated diarrhoea in swine has been scarcely studied.

Even though clinical signs typical of RV enteritis were successfully reproduced using gnotobiotic [23], as well as conventionally reared piglets [24], the virus was often regarded as low pathogenic to pigs. Nevertheless, recently, there has been an increasing number of peer-reviewed studies reporting cases of RVB-associated diarrhoea in pig herds in different countries [25–27]. Among the diarrhoeal samples from the USA, Canada, and Mexico tested for RVs 31.8% (2388/7508) were found RVB-positive [21]. Another study based on the material originating from the USA reported 46.8% (81/173) of samples positive for RVB with the highest detection rate noted in animals older than 55 days (72.7%; 16/22) [28]. Publicly available European data on the prevalence of RVB remain scarce and report extremely variable detection rates [24, 29].

In neonatal piglets, protection against RVs infection is narrowly restricted to the gut mucosal level and virus-neutralising IgA passively provided with milk from immune sows [20, 30]. In older pigs, the antibodies are produced by the gut-associated lymphoid tissues (GALT). Nevertheless, salient features of RVs such as vast genetic diversity, resistance to environmental factors and commonly applied disinfectants, no cross-protection between the species and poor-to-moderate cross-protection between serotypes, relatively low infectious dose, a short incubation period, and high titres of the virus excreted by affected piglets collectively limit the success rate of management strategies implemented at sow farms.

Despite the fact that rotavirus-associated diarrhoea is common in suckling piglets worldwide, there has been only a limited number of reports describing the role of RVB. Accordingly, very few sequences of the virus are publicly available. In order to gain a better insight into a porcine RVB enteritis case, this study aimed at the demonstration of the first molecular detection of the virus in samples collected from clinically affected suckling piglets in Poland employing Next Generation Sequencing followed by phylogenetic analysis of the obtained strain.

## Methods

### Study farm characteristics

The investigation was conducted in April 2024 in a Polish commercial, high-performing farm with 3,300 sows weaning piglets in weekly batches. All the animals were reared on a slatted floor following an all-in all-out system under conditions complying with the legal welfare requirements of Council Directive 2008/120/EC of 18 December 2008 laying down minimum standards for the protection of pigs. The herd was *Mycoplasma hyopneumoniae*-positive, toxigenic *Pasteurella multocida*-negative, *Actinobacillus pleuropneumoniae*-negative, porcine reproductive and respiratory syndrome virus (PRRSV)-negative, and *Brachyspira hyodysenteriae*-negative. All the pregnant sows and gilts were actively immunised prior to the farrow using Suiseng Coli/C and Suiseng Diff/A (Laboratorios Hipra S.A., Amer, Spain) administered according to the manufacturer’s recommendations.

### Key performance indicators

The production data were obtained from a commercial pig production management system implemented at the farm (Cloudfarms; Cloudfarms AS, Bratislava, Slovakia). The average number of piglets born alive and stillbirths per litter in a period of 31 days preceding the day of sampling was 18.39 and 1.57, respectively. The average weaning weight after four weeks of lactation was 5.96 kg. In the same timespan, the preweaning mortality rate was 16.30%, with low birth weight (< 600 g), crushing (overlying), and runts (including diarrhoeic piglets) reported as the main reasons of piglets death, accounting for 36.40% (585/1607), 34.41% (553/1607), and 24.21% (389/1607) of dead individuals, respectively.

### Clinical information

The exact number of litters and individual piglets clinically affected by the diarrhoea was not possible to evaluate due to cross-fostering procedures and a lack of ear tags with individual numbers allowing full traceability. According to the data provided by a farm manager and a veterinarian taking care of the herd, a diarrhoea incidence rate at the farm in a month preceding the day of sampling varied from 20% to 30% of piglets, with the vast majority of animals (> 75%) affected within their first week of life. All the clinically affected piglets were medicated with oral doxycycline (10 mg/kg b.w.; Doxycyclinum 200 Biofaktor, Biofaktor Sp. z o.o., Skierniewice, Poland) and the case-fatality rate (CFR) was reported to reach from 5% to 15%, what accounts for 1% to 5% mortality rate in an entire population of suckling piglets reared at the location. The surviving individuals had significantly reduced weight gain.

### Diarrhoeal samples

For the scope of this investigation, five 3-day-old dead diarrhoeic piglets (1195 g, 1605 g, 1240 g, 1095 g, 1425 g) were collected by a veterinarian and assigned numbers from 1 to 5. All the animals originated from different litters displaying clinical signs regarded as typical of rotaviral enteritis (watery diarrhoea) and were found dead due to crushing by a sow. Perinatal antimicrobial intervention had not been administered to the piglets. The carcasses were stored at -19 °C overnight and transported on the following day to SAN Group Biotech Germany GmbH (Höltinghausen, Germany) ensuring cold chain conditions. Ethical review and approval were waived for this study, as the analysed material originated from a routine diagnostic procedure ordered by the farm owners.

### Gross pathological examination of the digestive tract

The gross pathological examination of the digestive tract was conducted by a veterinary pathologist after thawing of the carcasses using a standardised scheme. Each animal was assessed for the filling state and gross pathomorphological lesions of the stomach, small intestine, and large intestine. Additionally, evaluations of consistency and colour of the contents were conducted specifically for the small and large intestines.

### Bacterial isolation

Samples for aerobic culture were collected from the small and large intestines of each animal. The obtained swabs were streaked out using a quadrant streaking method and inoculated onto six different agar plates (blood agar, BROLACIN agar, blood agar with neomycin, blood agar with gentamycin, McConkey agar and Slanetz-Bartley agar for *Enterococcae*) and incubated aerobically at 37 °C up to 72 h. The plates were examined macroscopically for growth every 24 h. Additionally, a *Clostridium* enrichment procedure was performed on all animals using meat broth and GC medium to provide optimal growth conditions for both *Clostridium perfringens* and *Clostridioides difficile.* Gained isolates of *Cl. perfringens*, *E. coli* and *Cl. difficile* were used for further typing.

### MALDI-TOF MS bacterial identification

In cases of positive growth, the gained isolates were confirmed using matrix-assisted laser desorption ionization-time-of-flight mass spectrometry (MALDI-TOF MS) performed on a MALDI Biotyper (Bruker Daltonik GmbH, Bremen, Germany).

### Polymerase chain reaction (PCR)

For the PCR analyses, the intestines (both the small and large) of individual piglets were pooled and examined separately for each animal. DNA and RNA extraction of the enteric samples was performed by routine molecular biological methods using the Kylt^®^ (SAN Group Biotech Germany GmbH, Höltinghausen, Germany) RNA/DNA purification kit. Real-time screening PCRs for Rotavirus A and C (Kylt^®^ porcine/bovine Rotavirus type A/Kylt^®^ porcine Rotavirus C), Porcine Epidemic Diarrhoea Virus and Porcine Transmissible Gastroenteritis Virus (Virotype^®^ PEDV/TGEV FLI-C 001) were performed according to the manufacturers’ instructions. Extraction as well as PCR Set up were processed fully automated in a high throughput laboratory setting. Culturally gained isolates were typed by PCR as follows: *Clostridium perfringens* major and minor-toxin genes and *Clostridioides difficile* toxins A and B were detected using real-time PCRs according to the manufacturers´ instructions (Kylt^®^ Clostridium perfringens, Kylt^®^ Clostridioides difficile Toxin A/B). *E. coli* virulence factors were also assessed using real-time PCR and covered 19 virulence factors (Kylt^®^ EAST, AIDA, paa, Kylt^®^ F4, F5, F6, Kylt^®^ FimA, FimH, F41, Kylt^®^ Sta, Stb, LT; in house methods for papC, eae, CdtB, cnf1, iucD, pic, escV).

### Next generation sequencing (NGS)

Total DNA and RNA were extracted from pooled intestinal samples (small intestine, ileum, colon) of all five piglets using the Kylt^®^ RNA / DNA Purification Kit (SAN Group Biotech Germany, Höltinghausen, Germany) according to the manufacturer’s protocol. Extracted samples are first treated with dsDNase according to the manufacturer’s instructions to remove contaminating genomic DNA. After DNase inactivation, the samples undergo host rRNA depletion. For this, custom-designed, host-specific DNA oligonucleotide pools are added to the RNA and hybridized under appropriate conditions, followed by enzymatic cleavage using thermostable RNase H. Following rRNA depletion, DNase is applied to degrade residual oligonucleotides. Each enzymatic step is followed by a purification using RNAClean XP Beads (Beckman Coulter Indianapolis, IN, USA) as per the supplier’s protocol to remove enzymes, buffers, and unwanted nucleic acid fragments. The resulting RNA is eluted in nuclease-free water and quantified for further downstream applications. Libraries for untargeted metagenomic sequencing were prepared using the Watchmaker RNA-Library Prep Kit (Watchmaker Genomics, Boulder, CO, USA) following the manufacturer’s instructions. The final library pool was sequenced on an Illumina MiSeq™ platform (Illumina, San Diego, CA, USA) using the MiSeq™ Reagent Kit v2 (300-cycles) (Illumina, San Diego, CA, USA). The subsequent data analysis and evaluation was performed by Base2Bio (Oshkosh, WI, USA).

### Phylogenetic analysis

The sequences were individually compared to sequences in GenBank by BLAST. A reference set of sequences was downloaded and used to assess the identity of the virus present in the samples using Geneious 10.2.6 (Biomatters Limited, Auckland, New Zealand). The sequences were aligned using MUSCLE and phylogenetic trees were reconstructed using maximum likelihood method with PHYML, using JC69 genetic substitution model [31, 32]. The tree topologies were verified by performing 1000 bootstrap replicates.

## Results

### Gross pathological examination of the digestive tract

The stomachs of all the sampled piglets were highly filled with coagulated milk and exhibited no gross pathological findings. The small intestines of four animals (1–4) were without macroscopic pathological manifestations. In one piglet (5) distention of the bowel loops was observed. The small intestines of piglets 1–4, and 5 were well and moderately filled, respectively. The content of the small intestine was described as pasty (1–4) or runny (5), and yellowish (1 and 3) or whitish (2, 4, 5). The large intestines of all the examined animals were without gross alterations, described as well (1–4) or moderately filled (5) with content characterised as pasty (1–3) or runny (4, 5), and yellowish (1–4) or whitish (5).

### Bacterial isolation

All the parameters evaluated prior to the non-directional sequencing procedure are demonstrated in Table 1. *E. coli* was isolated from all the samples. *C. perfringens* type A was cultured in 80.0% (4/5) samples. Samples obtained from 60.0% (3/5) piglets were *C. difficile*-positive.

**Table 1:** Bacterial piglet neonatal diarrhoea factors evaluated prior to the non-directional sequencing

Pathogen	Parameter			Sample					
				1	2	3	4	5	
*Escherichia coli*	Genes encoding virulence factors^1^	Adhesins	papC	−	−	−	−	−	
			eae	−	−	−	−	−	
			F4 (K88)	−	−	−	−	−	
			F5 (K99)	−	−	−	−	−	
			F6 (987P)	−	−	−	−	−	
			F41	−	−	−	−	−	
			AIDA	−	−	−	−	−	
			FimA	+	+	+	+	−	
			FimH	+	+	+	+	−	
			PAA	−	−	−	−	−	
		Toxins	CdtB	−	−	+	−	−	
			EAST1 (astA)	−	−	+	−	−	
			LT (elt)	−	−	+	−	−	
			Sta	−	−	+	−	−	
			Stb	−	−	+	−	−	
			cnf1	−	−	−	−	−	
		Other factors	iucD	−	−	−	−	−	
			pic	−	−	−	−	−	
			escV	−	−	−	−	−	
*Clostridium perfringens*	Isolation			+	+	+	−	+	
	Toxovar			A	A	A	n/a	A	
	Genes encoding virulence factors	Toxins	Alpha (M)	+	+	+	n/a	+	
			Beta (M)	−	−	−	n/a	−	
			Epsilon (M)	−	−	−	n/a	−	
			Iota (M)	−	−	−	n/a	−	
			Beta II (m)	+	+	+	n/a	+	
			Entero (m)	−	−	−	n/a	−	
			Pore forming netB (m)	−	−	−	n/a	−	
*Clostridioides difficile*	Isolation			−	+	+	−	+	
	Genes encoding virulence factors	Toxins	A, B	n/a	+	+	n/a	+	

^1^ – suckling piglet specific, + – positive sample, − – negative sample, n/a – not applicable, papC - pilus associated with pyelonephritis, eae – attaching and effacing protein (intimin), F4 (K88) – subunit faeG of F4 (K88) fimbriae, F5 (K99) – subunit fanC of F5 (K99) fimbriae, F6 (987P) – subunit fasA of F6 (987P) fimbriae, F41 – F41 fimbriae, AIDA – intestinal autotransporter adhesin, FimA – type-1 fimbriae (A chain), FimH – type 1 fimbriae (D-mannose specific adhesin), PAA – porcine attaching and effacing virulence factor, CdtB – cytolethal distending toxin subunit B, EAST1 (astA) – heat-stable cytotoxin associated with enteroaggregative *E. coli*, LT (elt) – heat-labile enterotoxin, Sta – heat stable enterotoxin I, Stb – heat stable enterotoxin II, cnf1 – cytotoxic necrotising factor, iucD – aerobactin synthesis, pic – serine protease autotransporter, escV – secretion of toxins (type III secretion system), M – major toxin, m – minor toxin

### Bacterial virulence factors associated with diarrhoea in suckling piglets

From the total number of 19 genes encoding suckling piglet specific *E. coli* pathogenicity factors evaluated during the investigation, 80.0% (4/5) individuals were tested positive for at least two of them. 80.0% (4/5) of the samples were FimA- and FimH-positive. Among them, material obtained form one piglet was found positive for five out of six tested *E. coli* toxin genes, coding for the following toxins: CdtB, EAST1, LT, Sta, and Stb. All the isolates of *C. perfringens* type A were found to be enterotoxin alpha- and beta II-producing. All the *C. difficile* isolates were tested toxin A- and B-producing-positive.

### Viruses associated with diarrhoea in suckling piglets evaluated prior to the non-directional sequencing (NGS)

None of the tested samples was demonstrated to contain the genetic material of porcine coronaviruses (PEDV, TGEV) and RVs (RVA, RVC).

### Next generation sequencing (NGS)

The NGS data for the sample resulted in 510,867 reads, 510,843 after adapter trimming. Of the 510,842 reads, 981 were assigned at species level to RVB (strain GCZ04) during read classification. The *de novo* assembly created 11 contigs, with the least common ancestor (LCA) RVB. For 10 contigs an additional annotation to RVB segment 1–6 or 8–11 was possible. The first contig, determined to be segment 1 of RBV (27 to 3491, 99% coverage), scored a coverage of 33.0 with a length of 3467 bp. During classification it mapped best to KR052714.1 with 84.85% identity. The coverage of the contigs varies between 28 and 41 with the lowest coverage is scored by the contig, determined to be segment 10 with a coverage of 28.0 and a length of 749 bp and the highest by the contig, determined to be segment 5 with a coverage of 41.0 and a length of 1257 bp. The RVB sequences were deposited in GenBank under name RVB/Pig-wt/Poland/GCZ04/2024/G16P4 (GenBank accession numbers PQ824600- PQ824610). The remaining viral reads were annotated to porcine kobuvirus (PKV) (23 reads). Furthermore, the analysis revealed presence of the following taxa: Qubevirus faecium (334 reads), and Gaprivervirus (1 read), bacteriophages not relevant to the problem discussed in our study. Besides the viruses, 40 bacterial genera (with Enterobacteriaceae, Lactobacillaceae, and Fusobacteriaceae as three most prevalent taxa with 185,866, 105,439, and 57,743 assigned read count, respectively) represented by 46 unique species were identified, including those commonly associated with diarrhoea in suckling piglets (*E. coli and C. perfringens*), tested using traditional methodology described above). No parasites were identified.

### Phylogenetic analysis

**Fig. 1:**
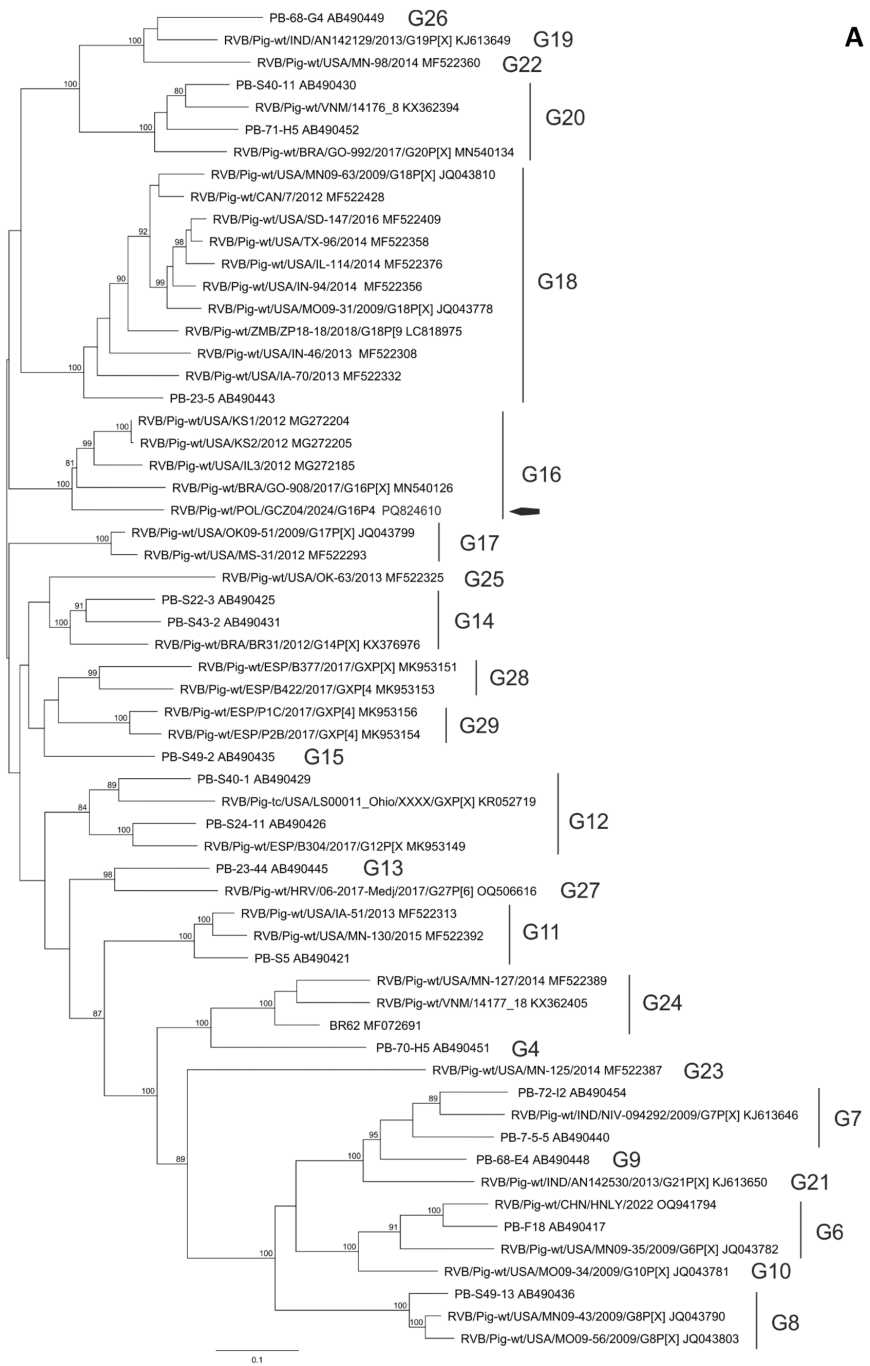

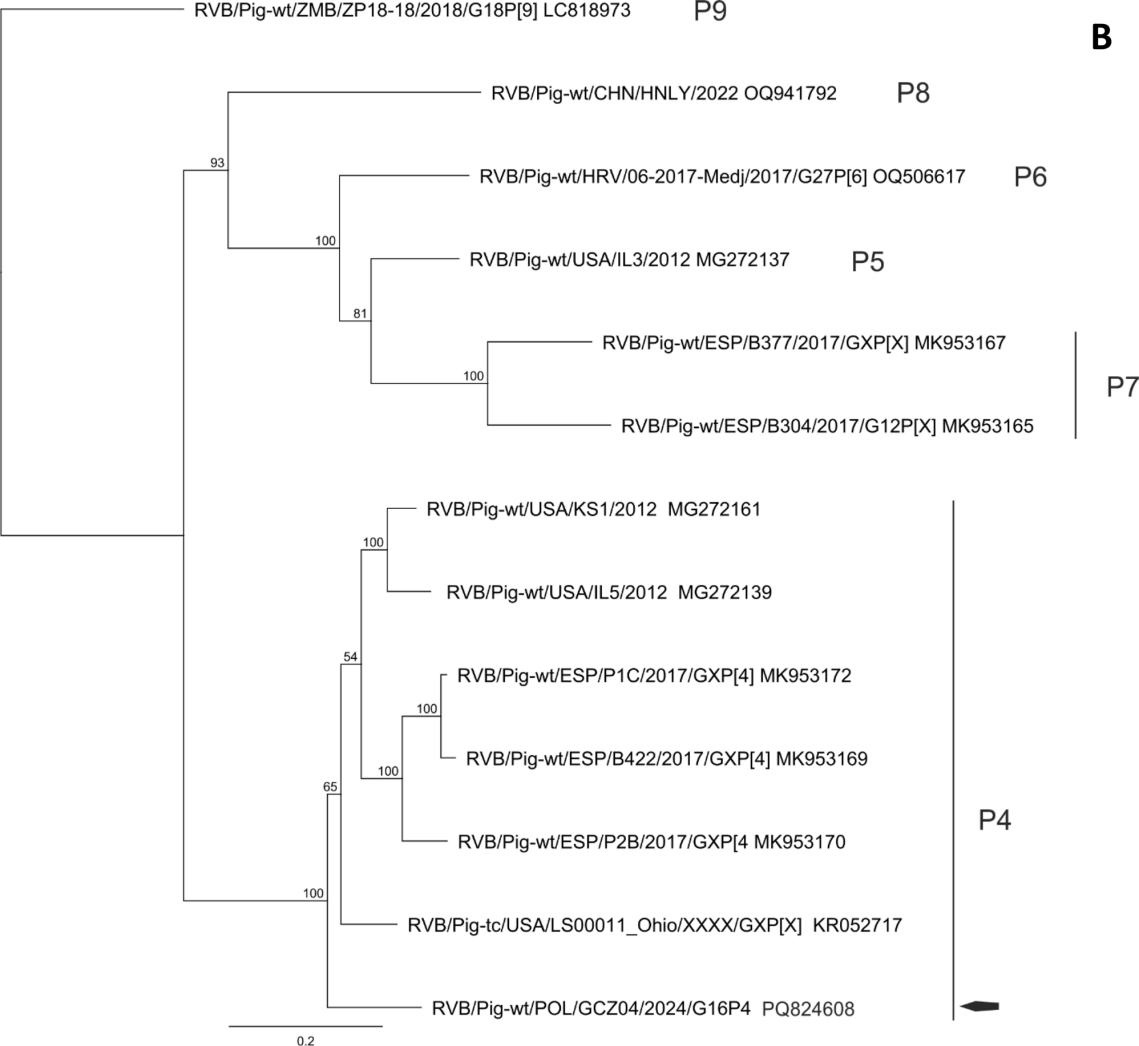
Phylogenetic trees constructed of selected nucleotide sequences coding for outer capsid proteins VP7 (**A**) and VP4 (**B**), using the maximum likelihood method with 1000 bootstrap replicates. Bootstrap values greater than 80 are shown. Only sequences of RVB of porcine origin were used for the analysis. A black arrow represents GCZ04 location in G16 and P[4] genotypes

The BLAST and phylogenetic analysis of the nucleotide sequences of the segments of GCZ04 strain coding for VP7, VP4, VP6, VP1, VP2, VP3 and NSP1-NSP5 of the RVB strain GCZ04 identified its genotype constellation to be G16-P[4]-I13-R4-C4-M4-A8-N10-T4-E4-H7. The percent identities of the nucleotide sequences of all segment were consistently above the cut-off values established for the genotypes [1]. The identity of all segment sequences to the most similar reference sequences from GenBank identified with BLAST search is presented in Table 2. Also, the phylogenetic clustering of the nucleotide sequences of the GCZ04 strain segments supported its genotyping (Fig. 1 and not shown).

**Table 2:** Genotype constellation of GCZ04 strain and the reference sequences with the highest nucleotide identity

Gene	Genotype	% nt identity	Strain	Acc. No.
VP1*	R4	84.85	RVB/Pig-tc/USA/LS00011_Ohio/XXXX/GXP[X]	KR052714.1
VP2*	C4	79.84	RVB/Pig-wt/ESP/B304/2017/G12P[X]	MK953181.1
VP3*	M4	83.17	RVB/Pig-tc/USA/LS00011_Ohio/XXXX/GXP[X]	KR052716.1
VP4	P[4]	83.65	RVB/Pig-tc/USA/LS00011_Ohio/XXXX/GXP[X]	KR052717.1
VP6	I13	87.67	RVB/Pig-wt/USA/IA09-67/2009/G16P[X]	KF882539.1
VP7	G16	84.87	RVB/Pig-wt/USA/KS09-44/2009/G16P[X]	JQ043791.1
NSP1	A8	86.6	RVB/Pig-wt/HRV/06-2017-Medj/2017/G27P[6]	OQ506622.1
NSP2	N10	85.98	RVB/Pig-wt/ESP/B304/2017/G12P[X]	MK953221.1
NSP3	T4	79.76	RVB/Pig-wt/USA/IL6/2012	MG272011.1
NSP4	E4	77.78	RVB/Pig-wt/RUS/KRSN5-2/2023	PP898332.1
NSP5	H7	83.62	RVB/Pig-wt/RUS/KRSN5-2/2023	PP898333.1

*Nearly complete sequences obtained

## Discussion

Despite its potential impact on the global swine industry, RVB still has not received much attention. Clinical research on its role in the development of diarrheal disease in pigs seem to be greatly hampered by the lack of RVB adaptation to tissue culture. Several additional factors, including frequent detection of the virus without its association with diarrhoeal disease, common coinfections with other members of the genus, and excretion of small quantities in stools of infected animals [33, 34] could have impaired systematic exploration of the issue as well. Nevertheless, commercially available modern technologies like NGS has proved useful in the identification and characterisation of novel pathogens in swine, including detailed exploration of multifactorial enteric diseases [35].

Our study reports the first molecular detection of porcine RVB in faecal samples collected from pigs reared on a commercial swine farm in Poland. Since the material was obtained from clinically affected animals and RVB was proven dominant among viral reads obtained during the NGS investigation [21], the virus can be considered as a potential causative agent of diarrhoeal disease in suckling piglets. Even though the detection of the virus has been well documented in several pig-rearing countries, comprehensive description of RVB-associated suckling piglets diarrhoea cases with the virus regarded as a primary causative agent is limited to few peer-reviewed papers published in recent years, namely reports from Russia [24], Brazil [36], and China [27], issued in 2018, 2020, and 2024, respectively.

The genotype constellation of GCZ04 was found to be typical for swine viruses and the nucleotide identities of the segments of the strain were within the cut off values established for different genotypes. The identities and phylogenetic clustering of different segment sequences to those from North America, Asia or Europe may suggest a complex evolutionary history of the Polish strain. However, more studies are needed to comprehend fully the evolution of European and globally distributed RVB strains from pigs. Full genome sequences of all the RVB segments are required to fully understand this virus’s evolutionary history. Unfortunately, the information on complete genome constellation of most strains is limited. Particularly from Eurasia, from where only eight Spanish, two Russian (from the Asian part), and one Croatian virus were sequenced in full [21, 37, 38].

The timing of the onset of diarrhoea described in these studies varied from one to seven days after birth what corroborates the timeframe noted in our investigation; however, only in the first two of the above-listed works, the diarrhoea was classified as watery. In the third one it was described as white and yellow cheese-like. Nevertheless, since unified criteria allowing unbiased comparison of porcine faeces structure have not yet been established and effectively implemented, such descriptions should be interpreted with considerable caution. Also, the gross pathological examination was carried out on unfrozen carcasses which could have induced deceptive alterations.

The morbidity rate observed in our study (20–30%) was markedly lower than these reported in all the previous cases, i.e. 60%, 30–50%, and 45%, in Russia [24], Brazil [36], and China [27], respectively. Also, the available case studies report highly variable mortality rates. The value of 1–5% dead piglets described in our study corresponds well with the rate recorded in a diarrhoea outbreak in China (3.8%). Slightly higher value, reaching 8%, was noted in Russia. At the other extreme there is a Brazilian research reporting a mortality rate varying between 10% and 50%. Nevertheless, the direct juxtaposition of these rates cannot be rationally justified, since various on-farm specific factors potentially affecting both, the spread of the virus and its clinical manifestation were not subjected to the investigations being discussed.

Morbidity rates could have been influenced by the level of passive immunity, internal biosecurity/hygiene procedures, or an average number of piglets born alive per litter forcing different cross-fostering procedures. Unlike previously published research, our study reported detection rates of coexisting bacteria and their virulence factors potentially affecting clinical manifestation of the disease. Similarly, even though all the samples analysed therein were tested negative for other known porcine diarrhoea-associated viruses, the available reports provided limited or no information on antibiotic medication of affected piglets, and ingredients of commercial/autogenous vaccines offered to pregnant dams. Therefore, mortality rates described in all the studies characterising the influence of RVB infection on affected suckling individuals cannot be attributed solely to the pathogenicity of a strain.

Frequent simultaneous detection of different RVs species has been well documented in swine [39, 40]. Nevertheless, conclusions regarding the clinical impact of the multiple infections on an affected individual brought therein remain highly ambiguous [27]. The issue of altered enteropathogenic potential of the viruses merits further discussion, since the exploration of archived samples collected at the study farm in July 2023 demonstrated the cooccurrence of RVs—RVA, RVB, and RVC were detected in 4.76% (1/21), 38.10% (8/21), and 38.10% (8/21) of ileal contents, and 0% (0/12), 66.67% (8/12), and 75.00% (9/12) of faecal samples collected from diarrhoeic piglets up to 7 days of age, respectively.

Prior to the NGS investigation, all the piglets sampled in our current investigation were tested negative for diarrhoea-associated viruses (RVA, RVC, TGEV, PEDV) using commercially available PCR setups. The only enteric virus of pigs found in NGS was PKV; however, based on a very low number of reads mapped for the virus (23 reads) and its unclear importance as a porcine pathogen, it can be reasonably inferred that PKV could not have been involved in the development of the diarrhoeal disease. Still, the reasonable interpretation of the role of RVB in the case reported in our study is largely obscured by the concurrence of several non-viral factors potentially exacerbating the clinical signs.

Among the set of genes coding for *E. coli* adhesins, four out of five samples were tested positive for two factors: fimA (type-1 fimbriae − A chain) and fimH (type 1 fimbriae − D-mannose specific adhesin), neither of which was a component of the vaccines offered to pregnant dams reared on the study farm. Even though commercially available vaccines containing fimbrial antigens of *E. coli* are commonly administered prior to the farrow, the set of adhesins remains narrowly restricted to those defined as the most prevalent worldwide, namely F4, F5, F6, and F41. Nevertheless, development of new licensed products targeting less obvious *E. coli* pathotypes affecting suckling piglets merits further investigation.

Additionally, one of the fimA- and fimH-positive samples was tested positive for five following *E. coli* toxins: cytolethal distending toxin subunit B (CdtB), heat-stable cytotoxin associated with enteroaggregative *E. coli* (EAST1), heat-labile enterotoxin (LT), heat stable enterotoxin I (Sta), and heat stable enterotoxin II (Stb), each of them reported in available scientific literature as diarrhoeagenic factors with a complex spectrum of biological activity.

Other toxinogenic intestinal bacteria strains isolated in the study involve toxin alpha- and beta II-producing *C. perfringens* type A, and toxin A/B-producing *C. difficile*. Since the set of commercial vaccines administered at the sow farm contains the components targeting only selected of the above-listed toxins, namely LT, *C. difficile* A and B toxoids, and *C. perfringens* type A alpha toxoids, the immunisation procedures could have provided the neonates with a relatively restricted level of passive protection. The quantification and evaluation of clinical relevance of neutralising antibodies following the active immunisation and/or natural exposure to the pathogens being discussed (including the leading role of RVB) was beyond the scope of our investigation.

Although the gross pathology of the digestive tracts described in our study was not clearly indicative of RV infection, it must be taken into consideration that all the intestinal samples were collected from naturally infected animals at the early stage of disease development. Also, it is noteworthy that the peer-reviewed data allowing direct and unbiased correlation between the severity of microscopic lesions caused by the RVs mono- or co-infections in suckling piglets and variability of clinical manifestation have not been published to date. For that reason, further research on the disease mechanisms, including histopathological examination carried out on RVB-affected intestinal epithelium will prove beneficial.

## Conclusions

To the best of our knowledge, this is the first study on the molecular detection of porcine RVB in clinical specimens collected from pigs in Poland. Despite limitations related to a relatively low number of sampled individuals and lack of histopathological examination performed on altered tissues, our results suggest that RVB should be routinely involved in a differential diagnostic procedure of the diarrhoeal disease in suckling piglets. Moreover, since the composition of commercially available vaccines stimulating development of adhesin- and toxin-specific antibodies did not correspond to the set of diarrhoea-inducing factors evaluated during the investigation, the potential role of various *E. coli* fimbrial adhesins and both clostridial and *E. coli* toxoids deserves deeper clinical examination.

## Data Availability

The datasets used and analysed during the current study are available from the corresponding authors on reasonable request.

## Abbreviations

AIDA: Intestinal autotransporter adhesin
CdtB: Cytolethal distending toxin subunit B
CFR: Case fatality rate
cnf1: Cytotoxic necrotising factor
dsRNA: Double-stranded RNA
eae: attaching and effacing protein (intimin)
EAST1: (astA) Heat-stable cytotoxin associated with enteroaggregative E. coli
escV: Secretion of toxins (type III secretion system)
F4: (K88) Subunit faeG of F4 (K88) fimbriae
F41: F41 fimbriae
F5: (K99) Subunit fanC of F5 (K99) fimbriae
F6: (987P) Subunit fasA of F6 (987P) fimbriae
FimA: Type-1 fimbriae (A chain)
FimH: Type 1 fimbriae (D-mannose specific adhesin)
GALT: Gut-associated lymphoid tissue
IgA: Immunoglobulin A
iucD: Aerobactin synthesis
LCA: Last common ancestor
LT (elt): Heat-labile enterotoxin
M: Major toxin
M: Minor toxin
MALDI-TOF: MS Matrix assisted laser desorption ionization-time of flight mass spectrometry
NGS: Next Generation Sequencing
NSP: Non-structural protein
PAA: Porcine attaching and effacing virulence factor
papC: Pilus associated with pyelonephritis
PCR: Polymerase chain reaction
PEDV: Porcine epidemic diarrhea virus
Pic: Serine protease autotransporter
PKV: Porcine kobuvirus
PRRSV: Porcine reproductive and respiratory virus
RV: Rotavirus
Sta: Heat stable enterotoxin I
Stb: Heat stable enterotoxin II
TGEV: Transmissible gastroenteritis virus
